# Imitation-mussel fluorescent silicon quantum dots for selective labeling and imaging of bacteria and biofilms

**DOI:** 10.3389/fbioe.2022.971682

**Published:** 2022-08-12

**Authors:** Jiayi Lin, Linlin Xu, Yuling Zheng, Dalin Wu, Jun Yue

**Affiliations:** Key Laboratory of Sensing Technology and Biomedical Instrument of Guangdong Province, School of Biomedical Engineering, Shenzhen Campus of Sun Yat-sen University, Shenzhen, China

**Keywords:** selective labeling, bacteria, bacterial biofilm, fluorescence, silicon quantum dots

## Abstract

Selective labeling of distinct bacteria and biofilm is poised for the fundamental understanding of bacterial activities, interactions, and coupled phenomena occurring at the microscale. However, a simple and effective way to achieve selective bacterial labeling is still lacking. Herein, we report a fluorescence probe with core-shell nanostructure that has polydopamine (PDA) coating on the surface of fluorescent silicon quantum dots (SiQDs@PDA). The surface of the SiQDs@PDA can be functionalized by various molecules (2-mercaptoethylamine hydrochloride, PEG, d-alanine, glucose amide) through different strategies (Michael addition, π-π interaction, and ion–ion interaction). Importantly, the d-alanine (D-Ala)- and gluconamide (Glc)-functionalized SiQDs@PDA fluorescence probes are capable of selectively labeling gram-positive and gram-negative bacteria, as well as their biofilms. The excellent performance in universal functionalization and selective labeling and imaging of bacteria and their biofilms demonstrate that SiQDs@PDA are a promising fluorescence tool in microbe research.

## Introduction

Innumerable bacterial species constitute a major part of the microbial ecosystem. Some of the bacteria play a positive role in our daily lives, while a large population of bacteria are threatening to our public health due to their toxicity, high vitality, and fast propagation. Selective labeling and imaging of living bacteria can promote the understanding of cellular heterogeneity in bacterial populations and advance the fast detection of bacterial species for effective treatments (Mao et al., 2018; Kwon et al., 2019; He et al., 2020; Ye et al., 2021; Zhang et al., 2021).

Gram staining is the gold standard method to label and classify bacteria based on the structural differences in the bacterial cell wall. However, this method involves complicated procedures and can only provide average information on the bacterial population. Meanwhile, many molecular detection methods such as surface-enhanced Raman scattering (SERS) (Zhao et al., 2022), polymerase chain reaction (PCR), and DNA sequencing technology (Shendure et al., 2017) have been developed, while the application of these technologies is limited due to the need of sophisticated equipment. To date, the most widely used method for bacterial labeling and imaging is fluorescence probe–based approaches such as flow cytometry and fluorescence microscopy. Compared with other technologies, fluorescence imaging is faster, is more economical, and can provide more direct and detailed information about bacteria. To achieve selective imaging and the classification of bacteria, the applied fluorescence probe should possess the ability to recognize different species. The heterogeneity in cellular composition, metabolites, and structure of bacteria are ready to be utilized for guiding the design of the fluorescence probe. For example, Kwon et al. (2019) reported a small molecular fluorescent probe of boronic acid–functionalized BODIPY. It could selectively label and image saccharide-rich gram-positive bacteria due to the specific interaction between boronic acid and the saccharide. Nonetheless, organic fluorescence probes suffer from low stability upon long-term irradiation (Zheng and Lavis, 2017; Demchenko, 2020). The development of new types of fluorescence probes with preeminent structural stability and high fluorescence efficiency for bacteria and biofilms is thereby in great demand.

Recently, semiconductor quantum dots, carbon nanodots, and fluorescent silicon quantum dots (SiQDs) have emerged as a new generation of fluorescence materials for their excellent stability and biocompatibility (Gao et al., 2018; Kasouni et al., 2019; Sivasankarapillai et al., 2019; Huang et al., 2020; Gao et al., 2021; Li et al., 2022; Martynenko et al., 2022; Özbilgin et al., 2022; Zhao et al., 2022). Among these, zero-dimensional SiQDs with high fluorescence quantum yield and narrow emission bands have received increasing attention for the sensing of biological and bacteriologic species. For example, the Lee group applied a one-pot microwave procedure to synthesize fluorescent SiQDs with great aqueous dispersibility and robust photostability and pH stability, which exhibited excellent fluorescence stability for long-term cellular imaging (He et al., 2011). To achieve the labeling of SiQDs onto specific bacterial species, it is necessary to functionalize the surface of the SiQDs by bioactive molecules such as peptides (Jung et al., 2021), proteins (Gidwani et al., 2021), and polysaccharides (Mozetič, 2019). However, such surface functionalization is problematic due to the ultrasmall diameter of the SiQDs (normally less than 10 nm) and a limited number of reactive groups on the surface of the SiQDs. Therefore, it is crucial to develop a universal method to simplify the modification procedure and advance the application of the SiQDs in bacterial imaging and labeling.

Inspired by the adhesion ability of mussels, the polydopamine (PDA)-coating technique has been widely used for the universal modification of nanostructures with bioactive molecules (Tian et al., 2019; Fu and Yu, 2020; Guo et al., 2020; Jing et al., 2020; Barros et al., 2021; Chen et al., 2021; Singh et al., 2021; Tian et al., 2021). This is achieved by versatile interactions which include hydrogen bond formation, covalent bond reconstruction, and π-π stacking interaction between PDA and bioactive molecules (Barclay et al., 2017; Schanze et al., 2018; Cheng et al., 2019; Patel et al., 2019). For example, Asha et al. (2022) reported a sugar-responsive coating system based on the reversible boronic ester bonds between catechol groups of PDA and benzoxaborole pendant of zwitterionic and cationic polymers, which displayed a promising self-cleaning function. d-alanine (D-Ala), one of the main components in the cell wall of gram-positive bacteria, can be directly integrated into the bacterial cell wall during metabolism (Hu et al., 2020; Mao et al., 2020), while gluconamide (Glc) is known for its strong interaction with lipopolysaccharide on the surface of gram-negative bacteria (Capeletti et al., 2019). Therefore, we hypothesized that SiQDs decorated with D-Ala and Glc could target gram-positive and gram-negative bacteria, respectively.

Herein, we report a new type of high-performance fluorescence probe with emission wavelength of 530 nm and fluorescence quantum yield of 44.7%, by applying the PDA-coating technique on SiQDs (SiQDs@PDA). The fluorescence probe SiQDs@PDA displays excellent adaptability for the functionalization of diverse molecules through the Michael addition reaction and supramolecular interaction. More importantly, low cytometry and fluorescence microcopy investigations have demonstrated that the D-Ala–modified and Glc-modified SiQDs@PDA are capable of selectively labeling gram-positive and gram-negative bacteria, respectively, as well as their corresponding biofilms. Compared with the small molecular fluorescent dyes which are prone to photobleaching under light irradiation, SiQDs-based probes developed in this study showed excellent stability against photobleaching and are of great potential in selective bacterial imaging.

## Results and discussion

### Synthesis and characterization of imitation-mussel–based fluorescent silicon quantum dots

**SCHEME 1:**
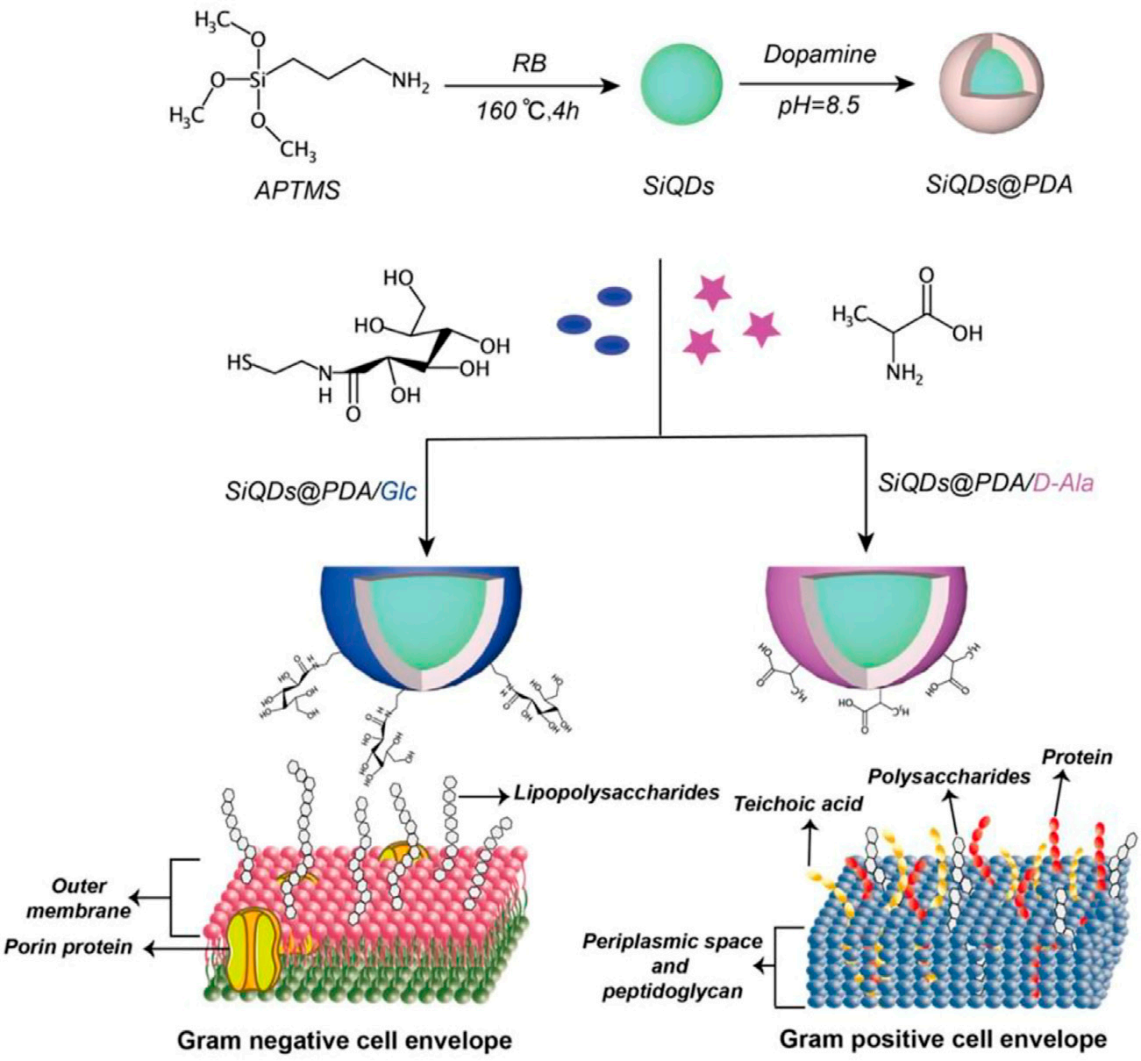
A schematic representation of the fluorescent probe preparation, surface functionalization, and selective bacterial labeling.

**FIGURE 1:**
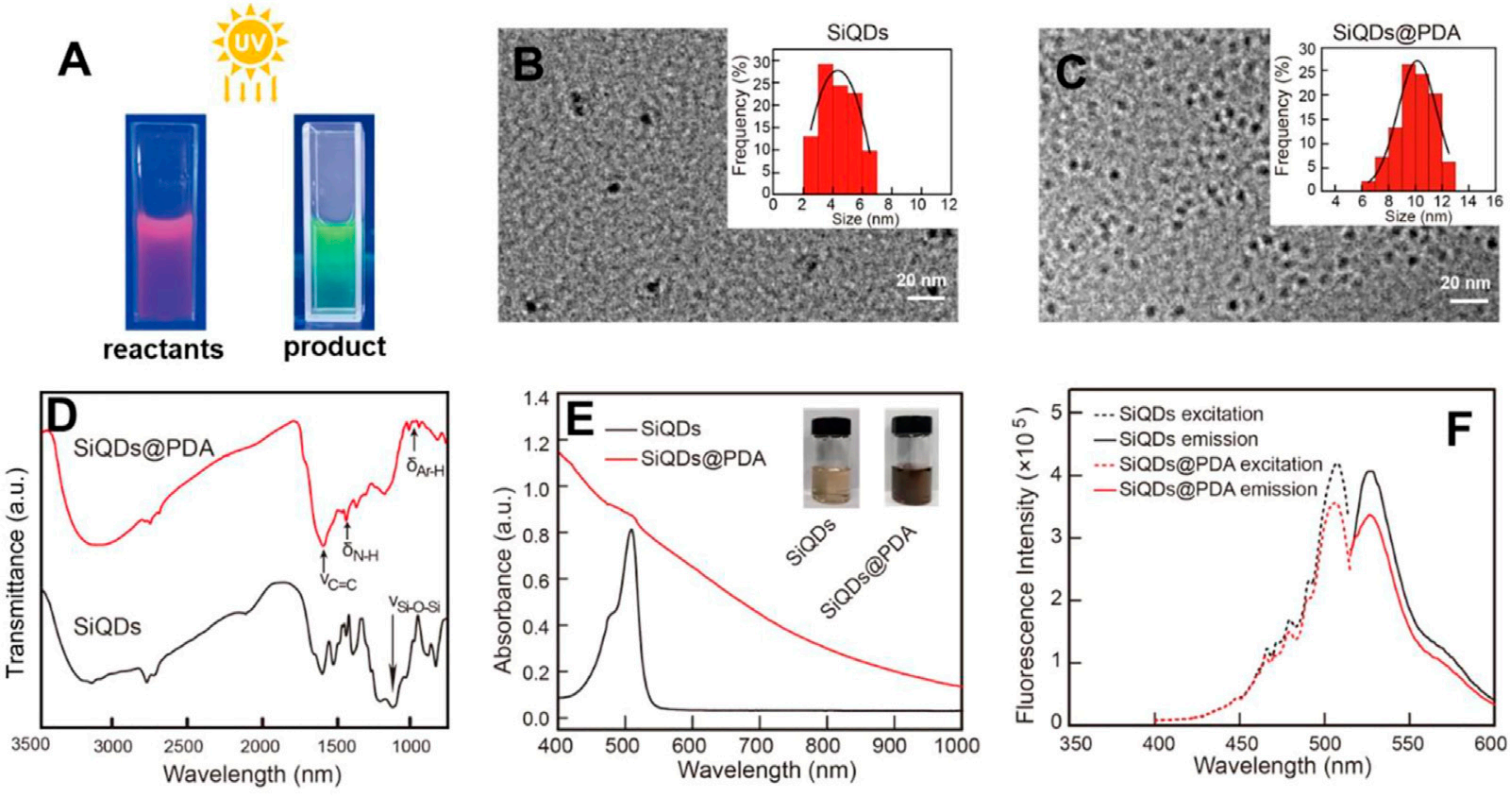
Characterization of SiQDs and SiQDs@PDA. **(A)** Digital images of the reactants in synthesizing the SiQDs fluorescent probe in aqueous solution under UV lamp irradiation; **(B,C)** TEM images with statistical analysis of average diameter; **(D)** FTIR spectra; **(E)** UV-Vis spectra; and **(F)** excitation and emission fluorescent spectra.

The fluorescent SiQDs were synthesized by the reduction of 3-aminopropyltrimethoxysilane (APTMS) or N-(2-aminoethyl)-3-aminopropyltrimethoxysilane (DAMO) under Rose Bengal (RB) *via* a one-step water–thermal reaction according to the published procedure (Chen et al., 2018), as shown in Scheme 1. The digital images of SiQDs aqueous solution under a UV lamp in Figure 1A and Supplementary Figure S1A clearly show the fluorescence signal demonstrating their fluorescence characteristic. Moreover, for both APTMS-based and DAMO-based SiQDs, the maximal emission and maximal excitation wavelengths of SiQDs are at 530 and 510 nm, respectively (Supplementary Figure S1B,C). As for the quantum yield (QY), APTMS-based SiQDs showed a slightly higher fluorescence QY (44.73%) than did DAMO-based SiQDs (41.02%, Supplementary Table S1), while they were all higher than previously reported carbon quantum dots and graphene quantum dots. Therefore, APTMS-based SiQDs were chosen for further research in the following sections.

The average diameter of SiQDs is 4 ± 1.6 nm according to the statistical analysis of the TEM image shown in Figure 1B. No aggregation is detected in Figure 1B, demonstrating that the SiQDs fluorescent probe has good dispersity in aqueous solution. In addition, the presence of transmittance peak with wavenumber 1100 cm^−1^ owing to -Si-O-Si- groups shown in Figure 1D verifies that SiQDs are constituted by the siloxane structure. Several publications have reported that the crystal structure in quantum dots normally influences the fluorescence characteristic (Liu et al., 2020; Terada et al., 2020). X-ray polycrystalline diffractometer (XRD) characterization indicates that the synthesized SiQDs had a feature of an amorphous state (Supplementary Figure S1D).

Subsequently, the fluorescent SiQDs@PDA were synthesized by simply mixing SiQDs with dopamine at pH 8.5 in Tris buffer at room temperature. According to the statistical analysis of the TEM image shown in Figure 1C, the average diameter of SiQDs@PDA is 9 ± 2.2 nm with a spherical nanostructure, and the increasing diameter after polymerization of dopamine indicates that PDA was successfully coated on the surface of the SiQDs. Likewise, no aggregation was observed in the TEM image, which demonstrates good dispersity of SiQDs@PDA in aqueous solution. In addition, the FTIR result in Figure 1D clearly shows new transmittance peaks with wavenumbers 1,610 cm^−1^ and 1,470 cm^−1^ owing to the stretching vibration of the benzene ring skeleton and bending vibration of the N-H bonds, respectively, and the relative strong transmittance peak at 990 cm^−1^ is attributed to the bending vibration of the Ar-H bonds in PDA, demonstrating the successful coating of PDA on the surface of SiQDs. Also, the UV-Vis spectra result shown in Figure 1E demonstrates that the absorption peak of SiQDs at 510 nm is almost masked by PDA for the fluorescent SiQDs@PDA probe. Finally, the fluorescence performance of SiQDs@PDA was investigated in depth. The excitation wavelength (510 nm) and emission wavelength (530 nm) of SiQDs@PDA remain unchanged when compared with those of SiQDs in aqueous solution (Figure 1F). It should be noted that the fluorescence intensity of SiQDs@PDA decreased slightly when compared to that of SiQDs, but the overall decline was less pronounced, which guarantees the subsequent fluorescence signal for labeling and imaging utilization in bacteria.

### Universal surface functionalization of imitation-mussel–based fluorescent silicon quantum dots

**FIGURE 2:**
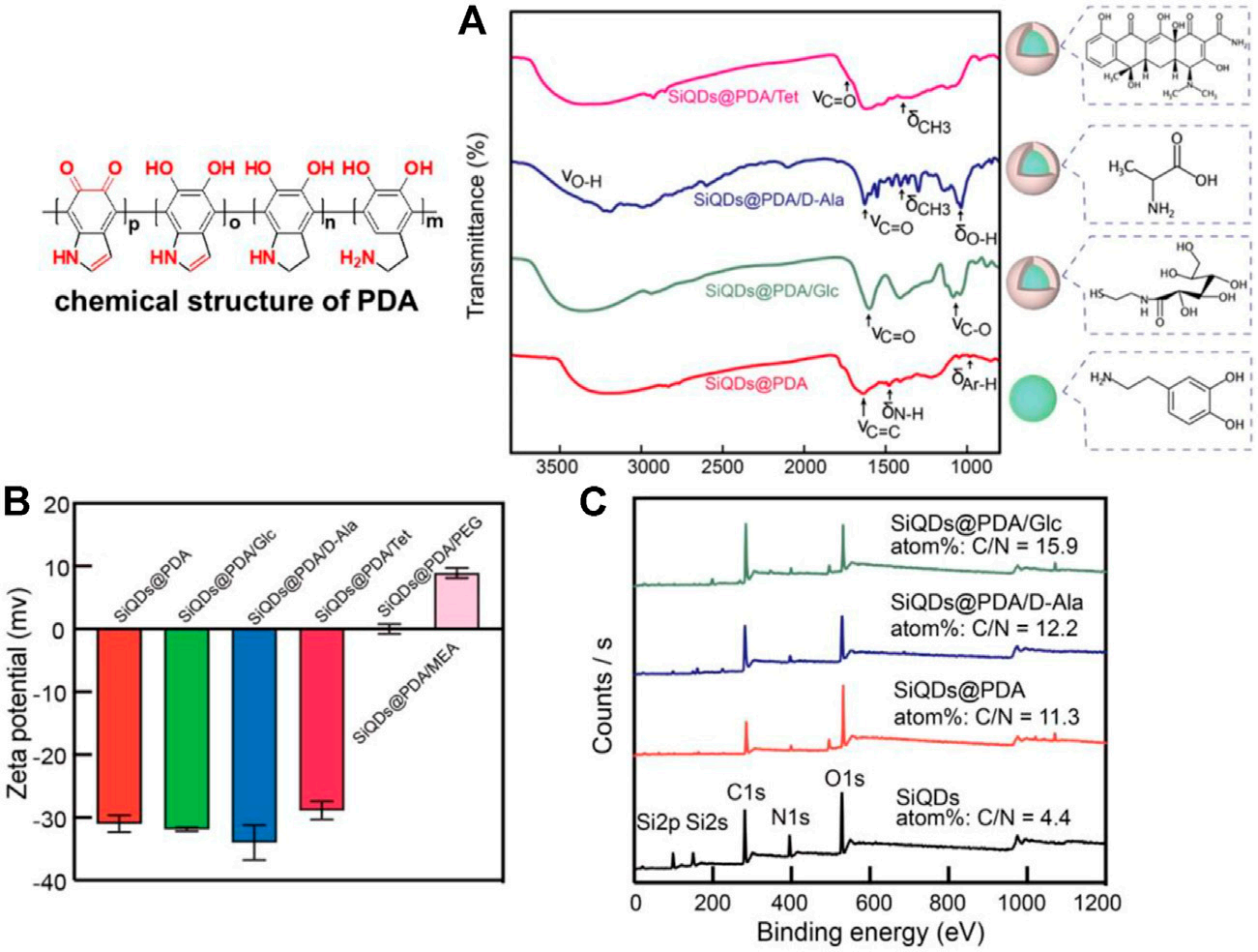
Characterization of functionalized SiQDs@PDA by various model molecules. **(A)** FTIR spectra of SiQDs@PDA after functionalization; **(B)** Zeta potential values of various model molecules–modified SiQDs@PDA in ultrapure aqueous solution; and **(C)** XPS results of D-Ala- and Glc-functionalized SiQDs@PDA.

Due to the existence of amine, hydroxyl, carboxyl, vinyl groups in PDA (Yang et al., 2020) as shown in Figure 2, in principle, various molecules could be modified on PDA by suitable chemistry strategies. Moreover, the negative charges and benzene rings of PDA facilitate PDA to form ion–ion and π-π interactions with specific ligands. To validate the surface functionalization universality of synthesized SiQDs@PDA, we selected three different types of molecules to modify the surface of SiQDs@PDA. The first reagent was tetracycline (Tet) containing several benzene rings in the molecule which is capable of being modified on the surface of SiQDs@PDA through π-π interaction. The second reagent was d-alanine (D-Ala) with positive charges that can adsorb onto the negatively charged PDA surface through ion–ion interaction. The third reagent was thiol-modified gluconamide (Glc), which could be coupled onto the surface of SiQDs@PDA by the Michael addition reaction.

First of all, FTIR was utilized to characterize the surface functionalization result; the corresponding results are presented in Figure 2A. Compared with the FTIR result of SiQDs@PDA (red solid line) shown in Figure 2A, the intense peak at 1,750 cm^−1^ is attributed to the stretching vibration of the C=O bonds in Tet, while the absorption peak at 1,430 cm^−1^ is attributed to the bending vibration of the saturated C-H bonds in Tet, demonstrating the successful coating of Tet on the surface of SiQDs@PDA through π-π interaction (pink solid line). Additionally, the broad and scattered peaks at 2,500–3,300 cm^−1^ is attributed to the carboxyl group of D-Ala; the relative strong transmittance peak at 1,600 cm^−1^ is attributed to the stretching vibration of the C=O bonds in D-Ala; the transmittance peak at 1,450 cm^−1^ is attributed to the bending vibration of the saturated C-H bonds; and the transmittance peak at 1,200 cm^−1^ is attributed to the bending vibration of hydroxyl groups of D-Ala (dark blue solid line). The presence of these typical peaks demonstrates the successful modification of SiQDs@PDA by D-Ala. For Glc, the relatively strong transmittance peak at 1,580 cm^−1^ is attributed to the stretching vibration of the C=O bonds in the amide groups, and the transmittance peak at 1,220 cm^−1^ is attributed to the stretching vibration of the C-O bonds of Glc, indicating that Glc was successfully modified on the surface of SiQDs@PDA by the Michael addition reaction (dark green solid line). In order to evaluate the universal validity of the Michael addition reaction for SiQDs@PDA surface modification, 2-mercaptoethylamine (MEA) (M_W_ = 77) and polymer PEG-SH (M_W_ = 2000) were chosen to modify the surface of SiQDs@PDA further. According to the FTIR result shown in Supplementary Figure S2, the strong transmittance peak at 2,984 cm^−1^ from the stretching vibration of the C-H bonds, the vibration transmittance peak at 1,110 cm^−1^ owing to the stretching vibrations of the C-O-C bonds in PEG (black solid line), the wide transmittance peak appearing near 3,392 cm^−1^ attributed to the −NH_2_ in MEA, and the transmittance peak at 1,166 cm^−1^ attributed to the stretching vibration of the C-N bonds in MEA (purple solid line) indicate that PEG and MEA were successfully coupled on the surface of SiQDs@PDA. Obviously, the Michael addition reaction in between thiol–vinyl is an effective strategy for SiQDs@PDA surface modification.

Besides FTIR, the zeta potential values of SiQDs@PDA after being functionalized by guest molecules were also systematically characterized to confirm the functionalization, and the results are presented in Figure 2B. Compared to SiQDs@PDA, the zeta potential values of Tet-, D-Ala-, and Glc-functionalized SiQDs@PDA do not change greatly and are still around −30 mV in ultrapure aqueous solution, which is attributed to the presence of many hydroxyl groups in Tet, D-Ala, and Glc. However, the zeta potential values of PEG and 2-mercapolamine (MEA) functionalized SiQDs@PDA are −0.1 mV and + 8.9 mV, respectively, indicating that the guest molecules have been successfully modified on the surface of SiQDs@PDA.

Among the above-tested molecules for surface functionalization, D-Ala belongs to bacterial metabolic activity molecules which mainly appear in gram-positive bacteria (Hu et al., 2020; Mao et al., 2020), and Glc is known for its strong interaction with lipopolysaccharide of gram-negative bacteria (Capeletti et al., 2019). We hypothesized that SiQDs@PDA after being functionalized by D-Ala and Glc could selectively label gram-positive and gram-negative bacteria, respectively. Before carrying out the selective labeling investigation, the coating results of D-Ala and Glc on the surface of SiQDs@PDA were further investigated by XPS. As shown in Figure 2C, compared with SiQDs@PDA, the ratio of C/N increased from 11.3 to 12.2 and 15.9 for SiQDs@PDA/D-Ala and SiQDs@PDA/Glc, respectively. The increase in the C/N ratio after D-Ala and Glc modifications on SiQDs@PDA is caused by higher C/N ratio values of D-Ala and Glc. In addition, the content value of the sulfur element is 1.05% for SiQDs@PDA/Glc. The elements analyses result indicates that SiQDs@PDA/D-Ala and SiQDs@PDA/Glc for bacterial selective labeling and imaging research were successfully prepared through the universal functionalization method.

### Selective labeling of SiQDs@PDA/D-Ala and SiQDs@PDA/D-Glc against bacteria

**FIGURE 3:**
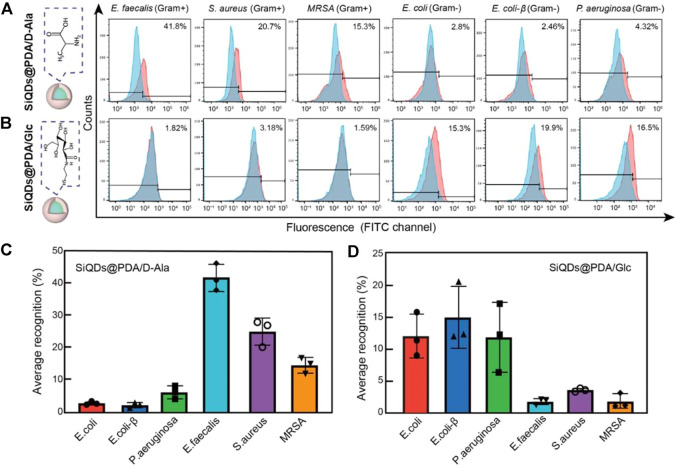
Gram-specific recognition of SiQDs@PDA/D-Ala and SiQDs@PDA/Glc. **(A,B)** Representative flow cytometry histograms collected from bacteria co-incubation with SiQDs@PDA/D-Ala and SiQDs@PDA/Glc. **(C,D)** Average recognition values for six different types of bacteria. Data = mean ± SD, *n* = 3.

In order to verify our hypothesis that SiQDs@PDA/D-Ala and SiQDs@PDA/Glc could selectively recognize gram-positive and gram-negative bacteria, respectively, SiQDs@PDA/D-Ala and SiQDs@PDA/Glc were cultured with six types of bacteria (three types of gram-positive bacteria: *Staphylococcus aureus*, *Methicillin-resistant S. aureus*, and *Enterococcus faecalis* and three types of gram-negative bacteria: *Escherichia coli*, *E. coli*-β, *Pseudomonas aeruginosa*) for 3 h. The flow cytometry results in Figure 3A show that after the co-incubation of bacteria with SiQDs@PDA/D-Ala, the 41.8, 20.7, and 15.3% portions of *E. faecalis*, *S. aureus*, and *MRSA*, respectively, are fluorescence stained, whereas the fluorescence signals of *E. coli*, *E. coli*-β, and *P. aeruginosa* are almost identical to the control bacteria, indicating that SiQDs@PDA/D-Ala could selectively label gram-positive bacteria. The statistical analysis in Figure 3C shows that the recognition ability of SiQDs@PDA/D-Ala to gram-positive bacteria varies significantly. *E. faecalis* possesses the highest recognition ability, followed by *S. aureus* and *MRSA*, which is probably due to the different thicknesses of the peptidoglycan layers for the three of them (Alkasher and Badi, 2020).

By contrast, as shown in Figure 3B, after the co-incubation of bacteria with SiQDs@PDA/Glc, 15.3, 19.9, and 16.5% portions of *E. coli*, *E. coli*-β, and *P. aeruginosa* were fluorescence stained, respectively, whereas the fluorescence signals of *E. faecalis*, *S. aureus*, and *MRSA* were almost identical to the control bacteria, demonstrating that SiQDs@PDA/Glc could selectively label gram-negative bacteria. Moreover, the statistical analysis shown in Figure 3D demonstrates that the recognition ability of SiQDs@PDA/Glc to the three tested gram-negative bacteria do not show significant differences, which is probably caused by the similar distribution of lipopolysaccharides on the outer surface of the gram-negative bacteria (Kim et al., 2016). Obviously, by functionalizing the surface of SiQDs@PDA by D-Ala and Glc, the selective labeling of gram-positive and gram-negative bacteria had been achieved successfully. Moreover, both SiQDs@PDA/D-Ala and SiQDs@PDA/Glc displayed negligible toxicity (Supplementary Figure S3), indicating that they are biocompatible in *in vivo* applications.

### Selective imaging of bacteria and biofilms with SiQDs@PDA/D-Ala and SiQDs@PDA/Glc as fluorescent probes

**FIGURE 4:**
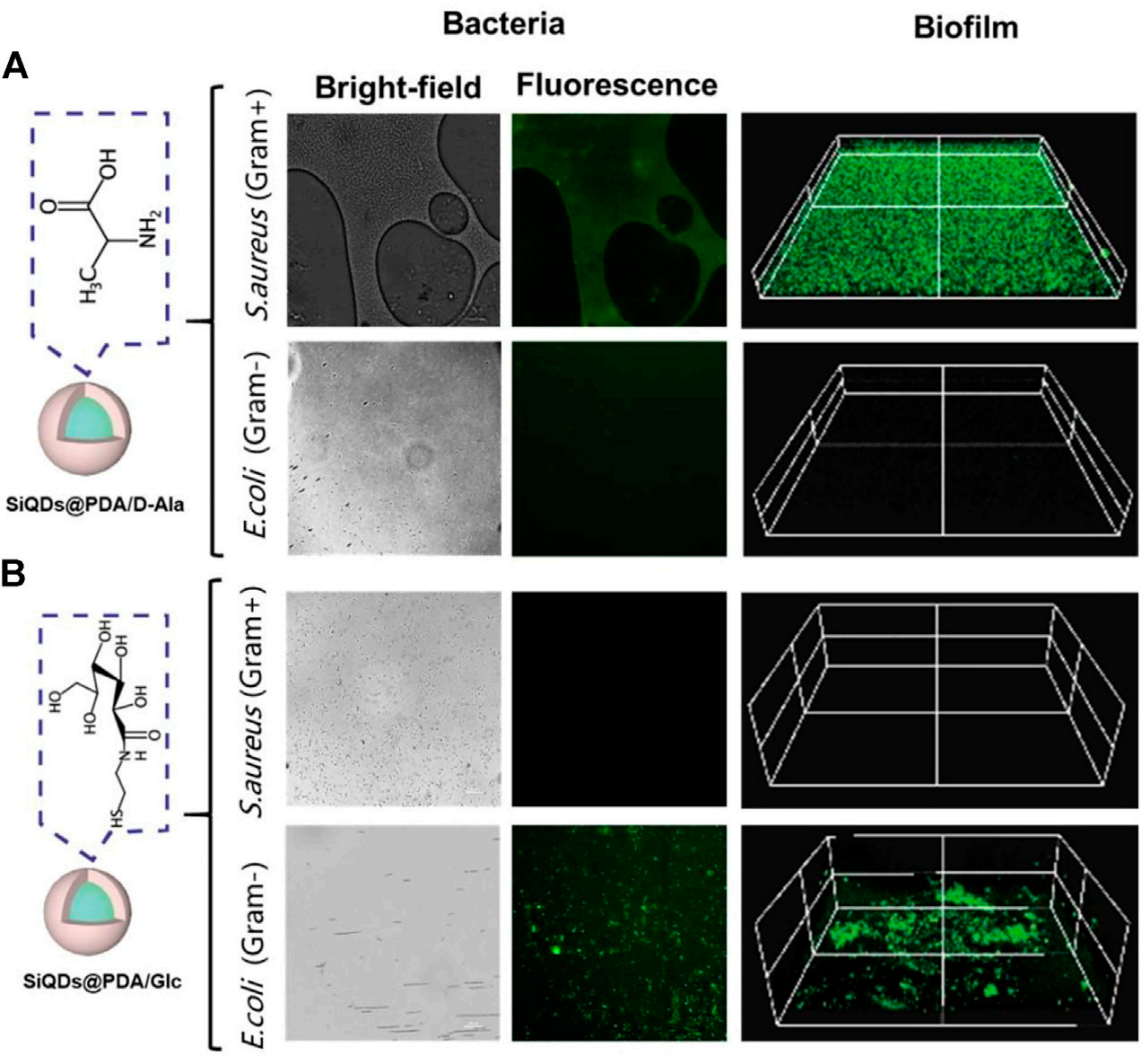
Characterization of selective imaging of bacteria and biofilms with SiQDs@PDA/D-Ala and SiQDs@PDA/Glc as fluorescent probes. **(A)** Optical and fluorescent imaging result of SiQDs@PDA/D-Ala as the probe and **(B)** optical and fluorescent imaging result of SiQDs@PDA/Glc as the probe.

The synthesized SiQDs exhibited an emission wavelength of 530 nm and 44.73% of fluorescence quantum yield. According to the flow cytometry result, SiQDs@PDA/D-Ala and SiQDs@PDA/Glc have a preference for staining gram-positive bacteria and gram-negative bacteria, respectively. As a result, SiQDs@PDA/D-Ala and SiQDs@PDA/Glc have the potential to be applied for selectively imaging gram-positive bacteria, gram-negative bacteria, and their biofilms as fluorescent probes. To verify the selective fluorescence imaging ability of SiQDs@PDA/D-Ala and SiQDs@PDA/Glc fluorescent probes, *S. aureus* and *E. coli* were chosen as model bacteria. After coculturing bacteria and their biofilms with SiQDs@PDA/D-Ala and SiQDs@PDA/Glc fluorescent probes, respectively, fluorescent microscopy was utilized to image the bacteria. As shown in Figure 4A, *S. aureus* showed strong green fluorescence, while *E. coli* had nearly no fluorescence under the same experimental procedure, which is consistent with the flow cytometry results, where SiQDs@PDA/D-Ala could selectively label gram-positive rather than gram-negative bacteria. In addition, the strong fluorescence signals in *S. aureus* biofilm and negligible fluorescence in *E. coli* biofilm indicate that SiQDs@PDA/D-Ala could not only selectively label gram-positive bacteria but also penetrate their biofilms. On the contrary, for fluorescent probe SiQDs@PDA/Glc, as shown in Figure 4B, the fluorescence signal only appears in *E. coli* and its biofilm, demonstrating that SiQDs@PDA/Glc could selectively label *E. coli* and penetrate through the biofilm unlike *S. aureus*.

**FIGURE 5:**
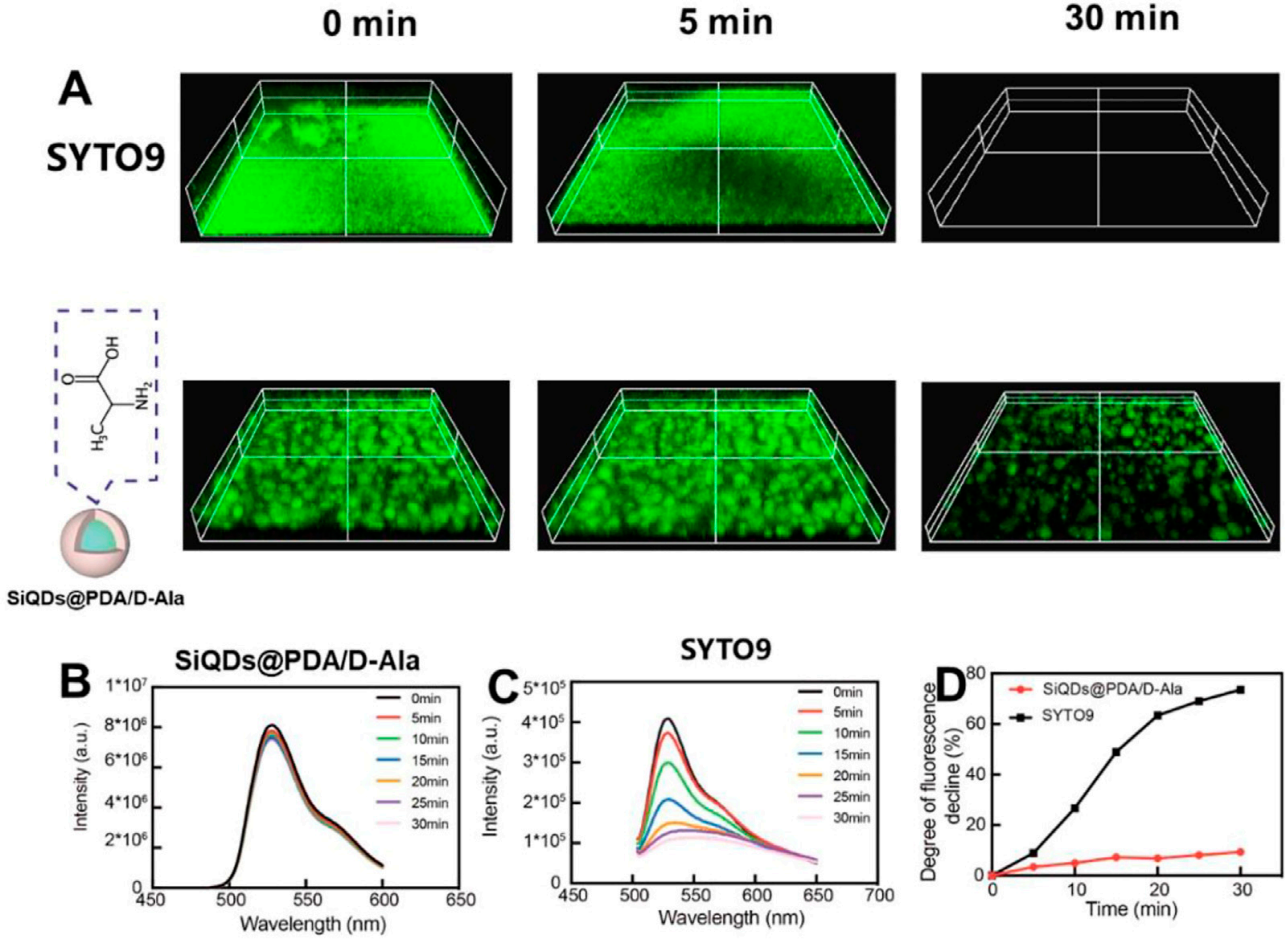
Anti-photobleaching characterizations. **(A)** Fluorescent images of SYTO 9 and SiQDs@PDA/D-Ala in *S. aureus* biofilm under white light irradiation; **(B)** fluorescent intensity of SiQDs@PDA/D-Ala under white light irradiation over time; **(C)** fluorescent intensity of SYTO 9 under white light irradiation over time; and **(D)** degree of fluorescent intensity decline of SiQDs@PDA/D-Ala and SYTO 9 under white light irradiation over time. The power of light is 2 W.

Next, we investigated the anti-photobleaching capacity of SiQDs, which is an important characteristic for bacterial imaging probes. The SYTO 9 dye, a widely used green-fluorescent nuclear and chromosomal counterstain permeant dye to both prokaryotic and eukaryotic cell membranes, was utilized to be compared with SiQDs. The images of the fluorescence microscope in Figure 5A show that the fluorescent signal of SYTO 9 in gram-positive bacteria biofilm disappeared after 30 min under white light irradiation (2 W). However, under the same conditions, SiQDs@PDA/D-Ala still showed strong fluorescent signal after 30 min. In addition, the fluorescent spectra show that the fluorescence intensity of SiQDs@PDA/D-Ala only decreased by less than 10% (Figures 5B,D) within 30 min under white light irradiation (2 W). While for SYTO 9, the fluorescent intensity decreased by about 73% (Figures 5C,D) under the same irradiation condition. Obviously, the synthesized SiQDs showed better anti-photobleaching properties than did the commercial organic fluorescent dye.

## Conclusion

The imitation-mussel fluorescent probe SiQDs@PDA with an emission wavelength of 530 nm and a fluorescence quantum yield of 44.7% was synthesized by *in situ* polymerization of dopamine on the surface of fluorescent silicon quantum dots. The universal functionalization ability of SiQDs@PDA was verified by successfully coupling various model molecules on its surface through π-π interaction, ion–ion interaction, and Michael addition reaction. On this basis, selective labeling and imaging of gram-positive bacteria, gram-negative bacteria, and their biofilms were achieved by modifying d-alanine and gluconamide onto the surface of SiQDs@PDA. Benefitting from the quantum confinement effect, SiQDs@PDA showed an excellent anti-photobleaching property. This imitation-mussel fluorescent SiQDs@PDA probe with excellent universal functionalization nature and anti-photobleaching performance is of great value for microbe-related research areas.

## Experimental section

### Materials

3-(aminopropyl)trimethoxysilane (Aladdin, ≥98%), rose bengal (Sigma-Aldrich, ≥80%), dopamine hydrochloride (Sigma-Aldrich, ≥99%), Tris base (Sigma-Aldrich, ≥99%), sulfhydryl-modified polyethylene glycol (PEG-SH) MW2000 (PEG2k-SH, Aladdin, ≥99%), 2-mercaptoethylamine hydrochloride (Sigma-Aldrich, ≥98%), tetracycline (Aladdin, ≥98%), d-alanine (Aladdin, ≥98%), tryptone soy broth (Solarbio Life Science, pH = 7.4), phosphate buffer (PBS, Gibco, pH = 7.4), sodium alginate (Aladdin, AR), gelatin (MW ∼50,000–100,000, type A from porcine skin), SYTO 9 (Thermo Fisher Scientific), *E. coli* (ATCC25922), *E. coli*-β (ATCC35218), *P. aeruginosa* (ATCC27853), *S. aureus* (ATCC6538), Methicillin-resistant *Staphylococcus aureus* (MRSA, ATCC43300) and *E. faecalis* (ATCC29212) were purchased from LuWei Tech. Co., Ltd., Shanghai, China.

### Synthesis of imitation-mussel florescent nanoprobe

*Synthesis of SiQDs.* According to the published method, SiQDs were synthesized with slight modification. The typical procedure is as follows: 20 mg of RB was dissolved in 8 ml of water first, then 2 ml 3-aminopropyltrimethoxysilane (APTMS) or N-(2-aminoethyl)-3-aminopropyltrimethoxysilane (DAMO) was added and stirred for another 5 min. The mixture was transferred to a 25-ml PTFE hydrothermal kettle and heated at 160 C for 4 h. After the temperature returned to room temperature, the liquid was transferred to a 500-Da dialysis bag to dialyze for 2 days. After that, the solution was rapidly frozen using liquid nitrogen for freeze-drying to obtain SiQDs powder.

*Synthesis of SiQDs/PDA.* 5 mg of SiQDs was added into 1 ml of water. Then, 0.4 ml SiQDs water solution was added into 9.4 ml Tris buffer (pH 8.5). Afterward, a 0.2 ml dopamine water solution (5 mg/ml) was added to this Tris buffer solution. After 1 h under stirring, the solution was transferred into a 1,000-Da dialysis bag for dialysis. Finally, brown SiQDs@PDA powder was obtained after lyophilization.

*Guest molecules functionalized SiQDs@PDA preparation.* Tet-, D-Ala-, Glc-, PEG-SH-, and MEA-functionalized SiQDs@PDA were prepared according to a similar procedure. The specific steps are as follows: 0.4 ml SiQDs water solution (5 mg/ml) and 0.2 ml dopamine water solution (5 mg/ml) were first mixed in 9.4 ml Tris buffer (pH = 8.5). Then, 6 mg of guest molecules were added to the aforementioned solution and stirred for another 2 h. Finally, the reaction medium was collected and transferred into a 3,500-Da dialysis bag and dialyzed against water for 2 days.

### Characterizations of florescent nanoprobes

TEM measurements of SiQDs and SiQDs@PDA were carried out by a JEM-2010HR instrument with an acceleration voltage of 200 kV. SiQDs and SiQDs@PDA fluorescent probes were diluted to 50 μg/ml with Millipore water. Then, 10 μl of each sample was dropped onto the copper grid and left to stand still in a ventilated dry place to dry the sample naturally. UV-Vis spectra of SiQDs and SiQDs@PDA were recorded using a UV-visible spectrophotometer (DU730) with a range of 400–1,000 nm.

The fluorescence characteristic of SiQDs was characterized by spectrofluorometer FS5. The concentration of the fluorescent probes was set at 70 μg/ml during the measurement, and the measurement temperature was set at 25 C.

Fourier transform infrared spectroscopy (FTIR) was recorded at wavenumbers between 650 cm^−1^ and 4,000 cm^−1^ on a Perkin Elmer Spectrum One Nicolet 520 spectrometer (United Kingdom) with 256 scans during the measurement. 10 mg SiQDs, SiQDs@PDA, and guest molecular functionalized SiQDs@PDA fluorescent probes were used for each measurement.

X-ray photoelectron spectroscopy (XPS) was recorded by ESCALAB 250 and Al K-Alpha X-ray as the excitation light source with a range of 0–1,200 eV and the energy level step width as 0.050 eV. The sample amount was maintained at around 10 mg per measurement.

XRD measurement was carried out by X’Pert PRO MPD instrument to investigate the crystallographic structure. The 2θ scan range was set at 30–90° under Cu Kα radiation (40 kV, 20 mA). The sample amount was maintained at around 10 mg per measurement.

The zeta potential values of different samples were measured by Malvern Zetasizer Nano ZS90. The sample concentration was set at 50 μg/ml in Millipore water, and the measurement temperature was set at 25 C.

### Evaluation of the functionalized silicon quantum dots in selective labeling of bacteria

In order to assess the selective labeling effect of SiQDs@PDA/D-Ala and SiQDs@PDA/Glc against bacteria, we selected gram-negative bacteria *S. aureus* and gram-positive bacteria *E. coli* to analyze the selective labeling effect on both bacteria by targeted fluorescence silicon quantum dots by upright fluorescence microscope (NI-U) and having the quantitative fluorescence intensity evaluated by flow cytometry (Beckman CytoFLEX).

The specific experimental steps for the qualitative analysis are according to the following steps: 1) after coculturing with *S. aureus* and *E. coli* overnight in sterile tryptone soy broth (TSB), 500 μl bacteria solution was taken into a 1.5-ml sterilized centrifuge tube and mixed with 700 μl SiQDs@PDA/D-Ala (70 μg/ml) and SiQDs@PDA/Glc (70 μg/ml) for 3 hours; 2) the bacterial mixture was centrifuged as the last step at 7,000 rpm for 5 min. The supernatant was aspirated and the bacteria resuspended in 100 μl sterile PBS, then 50 μl was dropped on the cleaned glass slide. The morphology and labeling results of the bacteria were characterized by using the upright fluorescence microscope (NI-U).

The experimental steps for the quantitative analysis were similar to the qualitative analysis. Three kinds of gram-positive or gram-negative bacteria were cocultured with SiQDs@PDA/D-Ala or SiQDs@PDA/Glc for 3 hours, respectively. The mixture was centrifuged at 3,000 rpm for 3 min and the supernatant was aspirated. The mixture was then washed with sterile PBS. Finally, the mixture was suspended in 500 μl PBS, and the FITC channel was chosen to observe the bacterial labeling by flow cytometry (Beckman CytoFLEX) while the flow rate was 30 μl/min and 10,000 bacteria samples were recorded totally.

### Evaluation of the functionalized silicon quantum dots in selective targeting bacterial biofilms

3D biofilm models of *E. coli* and *S. aureus* were constructed *in vitro* by using 3D bioprinting technology to further investigate the labeling of biofilms by the materials.

Firstly, 2.5% sodium alginate and 8% gelatin (Gel) were mixed in a beaker, heated at 50 C for 30 min, and then kept at 4 C for 5 min to remove air bubbles. After the heat–cool cycle had been repeated three times, the final mixture solution (Alg-Gel) was placed in a sterile syringe and incubated at 37 C for 20 min to form a complex colloidal solution.

Secondly, 10 μL of frozen *E. coli* or *S. aureus* was thawed and inoculated in 12 mL of sterile TSB medium respectively, followed by incubation in 37°C shaker overnight. The bacterial solution (OD600 = 0.3) was centrifuged at 3,000 rpm for 3 min and the supernatant was aspirated. The precipitated bacteria were resuspended in 500 μl of sterile TSB medium to obtain a concentrated bacterial suspension. The bacterial suspension was added to 10 ml of Alg-Gel solution and vortexed at 1,500 rpm for 5 min at room temperature to obtain a homogeneous bioink containing the bacteria.

Finally, the samples were printed using the EnvisionTEC 3D-Bioplotter. Before use, the bioplotter was sterilized by UV light and wiped with 75% ethanol. A sterile 10-ml syringe containing the bacterial bioink was attached to the print nozzle and loaded into the bioprinter. The bioink containing bacteria was dispensed through a needle with an internal diameter of 200 μm at a printing speed of 8–10 mm/s and an extruded air pressure of 1.2–1.4 Bar. 3D printed cubes were 15 mm in length and width and approximately 1 mm in height. After 3D printing, the constructs were cross-linked and cured in 20 mM BaCl_2_ solution for 5 min. The cross-cured constructs were placed in a sterile 6-well culture plate and washed carefully with sterile PBS. Then, PBS was aspirated and 3 ml of sterile TSB medium was added to the wells of the plate. This was then incubated at 37 C, 100 rpm and the TSB medium changed every 1–2 days to obtain a dense 3D biofilm after 14–16 days of incubation.

### Biocompatibility of silicon quantum dots, SiQDs@PDA, and ligand molecules–functionalized SiQDs@PDA

The cytotoxicity of SiQDs, SiQDs@PDA, and D-Ala- or Glc-modified SiQDs@PDA was assayed by MTT by using the A549 cell line. Briefly, 5 × 10^3^ cells were seeded in 96-well plates with 200 μl medium per well and incubated overnight. After 6 h exposure to 70 μg/ml SiQDs, SiQDs@PDA, SiQDs@PDA/D-Ala, and SiQDs@PDA/Glc, 20 μl of 5 mg/ml MTT was added to each well and incubation was continued again for 4 h. Finally, 200 μl DMSO was added to each well and the absorption at 490 nm was measured with a microplate spectrophotometer (Synergy Mx, BioTek).

## Data Availability

The original contributions presented in the study are included in the article/Supplementary Material; further inquiries can be directed to the corresponding authors.
