# Supplementary material for: Imitation-mussel fluorescent silicon quantum dots for selective labeling and imaging of bacteria and biofilms
Source: Front Bioeng Biotechnol. 2022 Aug 12;10:971682. doi: 10.3389/fbioe.2022.971682 (PMC9411794; doi:10.3389/fbioe.2022.971682)
Supplement: Supplementary file 1 [file Table1.DOCX]

**Supporting Information**

**Imitation-mussel Fluorescent** **Silicon Quantum Dots for Selective Labeling and Imaging of Bacteria and Biofilms**

Jiayi Lin^1†^, Linlin Xu^1†^, Yuling Zheng^1^, Dalin Wu^1*^, Jun Yue^1*^

*^1^Key Laboratory of Sensing Technology and Biomedical Instrument of Guangdong Province, School of Biomedical Engineering, Shenzhen Campus of Sun Yat-sen University, Shenzhen, Guangdong 518107, P. R. China.*

*^*^To whom correspondence should be addressed: yuejun3@mail.sysu.edu.cn; wudlin6@mail.sysu.edu.cn*

*^†^These authors contributed equally to this work.*


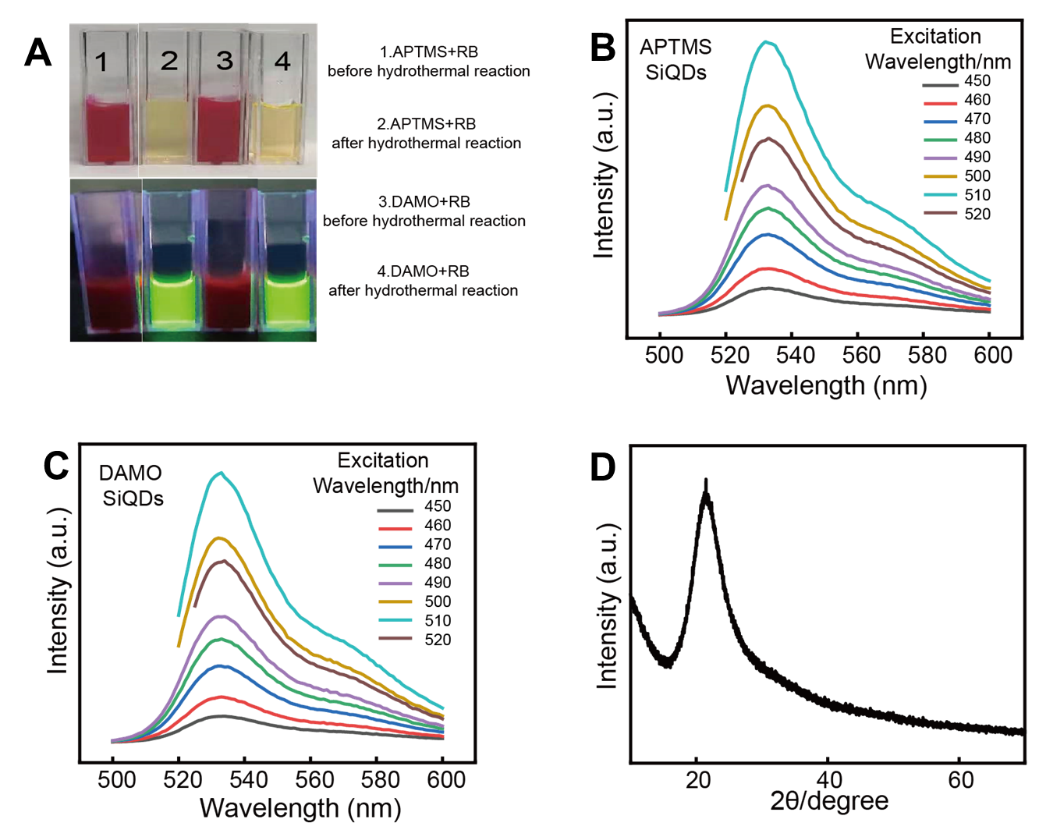
Figure S1. **APTMS and DAMO based SiQDs fluorescence properties and structure characterization.** (A) digital images of APTMS based SiQDs and DAMO based SiQDs before and after the reaction under white light and UV lamp, (B) fluorescence emission spectra of APTMS based SiQDs under different excitation wavelengths, (C) fluorescence emission spectra of DAMO based SiQDs under different excitation wavelengths and (D) XRD pattern of APTMS based SiQDs.

**
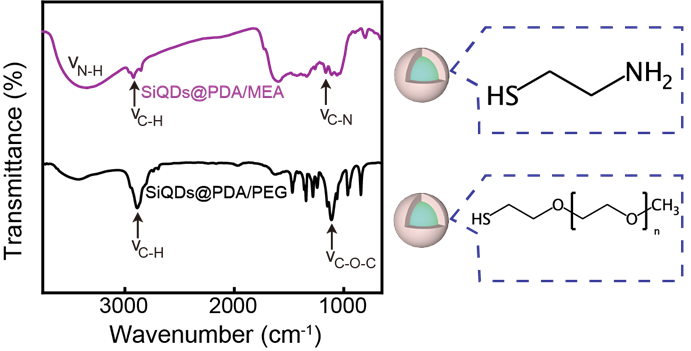
**

**Figure S2.** FTIR spectrum of 2-mercapolamine (MEA) (MW = 77) and polymer PEG-SH (MW = 2000) modified SiQDs@PDA through thiol-vinyl Michael addition reaction.

Table S1. **Fluorescence quantum yield of SiQDs obtained by different synthesis methods**

| **Synthesis**  **method** | **Silane coupling agents** | **Reducing agent** | | [**Reaction conditions**](https://www.baidu.com/link?url=Q1c37gVdfIoGXKIQKkG_a1B2OblFqx0KyIxBSQt6v9vcnr7LOiQlbqHJOj8aqPe8nCcoYZHAm2wpgjXtUVYYJHrFa5xbyixGQ2zEZrO_Utp_xeBmMFjfO12wK3ZIt7ig&wd=&eqid=a5d875a00002b334000000066229f8de) | **Ex/Em**  **(nm)** | **Absolute quantum yield** | |
| --- | --- | --- | --- | --- | --- | --- | --- |
| Hydrothermal method | 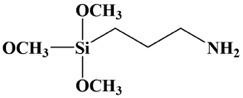 | | 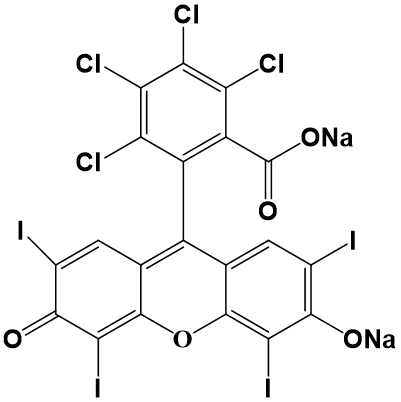  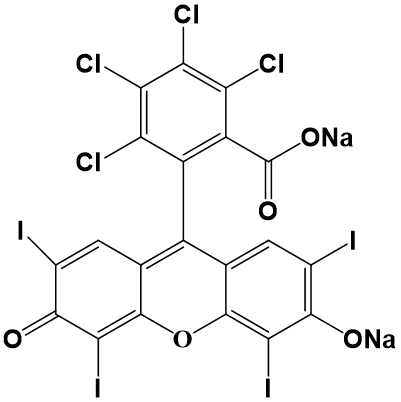 | 160℃ Hydrothermal | 510/530 | | **44.73%** |
| Hydrothermal method | 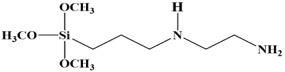 | |  | 160℃ Hydrothermal | 510/530 | | **41.02%** |


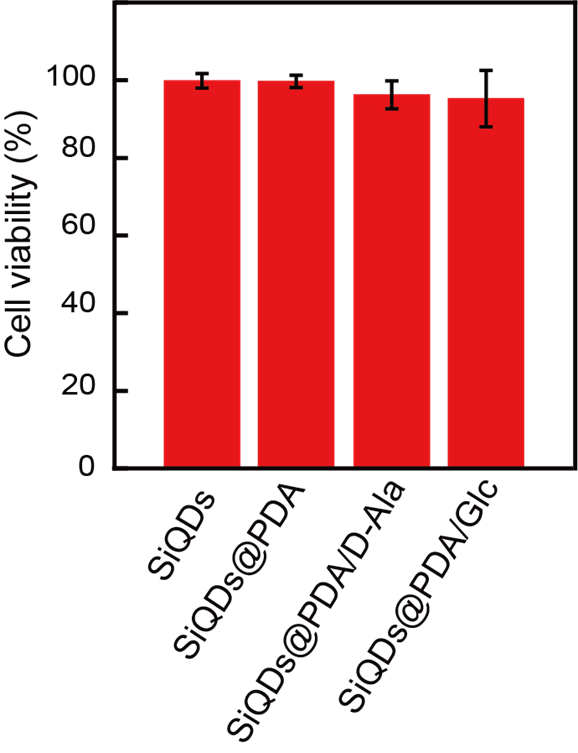


Figure S3. Biocompatibility characterization result of mentioned SiQDs probes in this article with concentration 150 μg/mL.
